# Isokinetic and Isometric Assessment of the Knee Joint Extensors and Flexors of Professional Volleyball Players

**DOI:** 10.3390/ijerph18136780

**Published:** 2021-06-24

**Authors:** Piotr Wilkosz, Jaroslaw Kabacinski, Krzysztof Mackala, Michal Murawa, John Ostarello, Agata Rzepnicka, Lukasz Szczesny, Anna Fryzowicz, Jacek Maczynski, Lechoslaw B. Dworak

**Affiliations:** 1 Department of Physiotherapy, Stanislaw Staszic University of Applied Science in Pila, ul. Podchorazych 10, 64-920 Pila, Poland; pwilkosz@tlen.pl; 2 Department of Biomechanics, Poznan University of Physical Education, Królowej Jadwigi 27/39, 61-871 Poznan, Poland; murawa@awf.poznan.pl (M.M.); fryzowicz@awf.poznan.pl (A.F.); maczynski@awf.poznan.pl (J.M.); 3 Department of Track and Field, University School of Physical Education, ul. Paderewskiego 35, 51-612 Wroclaw, Poland; krzysztof.mackala@awf.wroc.pl; 4 Department of Kinesiology, California State University, Hayward, CA 94542, USA; john.ostarello@csueastbay.edu; 5 Public Medical Center ‘Doktor Krasicki’ in Gdynia, ul. Zakręt od Oksywia 3, 81-244 Gdynia, Poland; rzepnicka.agata@gmail.com; 6 VeloLAB Physiotherapy Biomechanics Lukasz Szczesny, ul. Starodworska 3, 80-137 Gdansk, Poland; lukasz.szczesny@velolab.pl; 7 Faculty of Health Sciences, Calisia University, ul. Nowy Świat 4, 62-800 Kalisz, Poland; l.dworak@akademiakaliska.edu.pl

**Keywords:** muscle torque, isokinetics, statics, biomechanics, knee joint, volleyball

## Abstract

Purpose: The main objective of this study was to evaluate the level of muscle strength by using isokinetic and isometric measurements—more specifically, the force ratio between the knee flexors and extensors (values of the torques). Methods: An experimental group of elite volleyball players (n = 14) were compared to a control group (n = 14) of healthy non-athletes of comparable ages. Torque measurements were obtained under three concentric conditions (angular velocities of 60 °/s, 180 °/s, and 300 °/s) and one static condition by utilizing the Biodex System 3. Results: In all trials, the volleyball players achieved significantly higher peak torque (PT) values for both the extensors and flexors (*p* < 0.05) than those of the control group. However, the strength ratio of the flexors and extensors (H/Q) in the experimental group was only 83% of the standard reported in the literature. The most developed and dominating muscles in the knee joints of the volleyball players were the extensors, which accounted for the low strength ratio and dynamic instability of this joint. Conclusion: Based on a proper assessment of the strength ratio of the knee flexors and extensors, properly selected and implemented resistance training can improve the maximum strength and power production and reduce the incidence of injuries in volleyball.

## 1. Introduction

Volleyball is a non-contact team sport that requires players to alternately perform high-intensity actions involving passing a ball and scoring points against the opposing team on their court [1]. The ball-passing actions also involve jumping activities that are performed near the net (e.g., sets, spikes, and blocks) and a jumping serve that is executed at one end of the court. These movement structures are critical for executing such actions and have a great impact on the final game performance [2]. Additional factors for success in the game of volleyball are the quality of the jumps performed (vertical jump height), the technique of passing the ball, and the players’ anthropometry (height of the body). Moreover, the repetitive loading of the joints from the jumping and landing actions—often executed more than 200 times in one match [3,4,5]—exposes players to a high likelihood of injury [6,7]. A characteristic of this jumping and landing is an extreme impact overload that exceeds a player’s body mass by several times.

Knowing how many different jumps volleyball players perform during a game can provide new insight into the quality of their specific strength requirements. It can help design appropriate volleyball training and, most of all, monitor and control the improvement of training results [8,9]. Current technological advances have made it possible to control the level of muscle strength, especially the force ratio between the knee flexors and extensors, which helps in the assessment of aspects of the quality of the jumps and thus in determining the load on the knee joint [8,10,11,12]. Additionally, the second factor mentioned above can lead to different types of injuries. Statistics show that in volleyball, 60% of injuries occur during training sessions and 40% occur during competitions. Among the participants in this sport, injuries happen most frequently in the following joints: knees, ankles, shoulders, lumbar spine, and small joints of the hand [6,13,14]. Therefore, the disturbing force ratio between the knee flexors and extensors could cause an accumulation of microtraumas and then the spread of chronic excessive trauma, such as patellar tendinopathy [7,15].

Maintaining the ability to perform jumps with a qualitatively explosive force—which is defined as the ability of the neuromuscular system of an individual to manifest the load in the shortest possible time [16]—is considered one of the priorities in volleyball training [5]. Therefore, according to McNitt-Gray [17], landing from an increased height increases the mechanical demand and eccentric force of the impact, which directly increases the peak extensor joint moments in the ankle, knee, and hip. In turn, these requirements are related to the assessment of the level of a player’s muscle strength, which can be performed under isokinetic and isometric conditions [10,11,18]. In isokinetic testing, the resistance is adjusted to the strength of the individual while the angular velocity is fixed. This allows the measurement of the net torque of the knee extensors and flexors [10,11,19,20].

Dauty et al., [21] conducted tests on volleyball players on the French national team. The Cybex 6000 system was used to analyze the peak torque (PT) during concentric and eccentric work. The values achieved by the participants were slightly higher than the population standard for the hamstring/quadriceps ratio (H/Q)—0.66 vs. 0.60. These researchers suggested that under functional conditions, a better indicator of the balance between the knee flexors and extensors would be the ratio measured during the eccentric work of the flexors and the concentric work of the extensors. Bittencourt et al. [22] carried out tests on representatives of Brazil’s junior teams—aged 19 to 21—using the Biodex System 3. The strength H/Q ratio for this group was low—at the level of 0.51. Continuing this analysis, an interesting comparison of the strength of the knee joint flexors and extensors in volleyball players and soccer players was presented by Magalhaes et al. [23]. They found that the PT and H/Q ratios were only significantly lower in volleyball players at 90 °/s and that their lower limbs’ values were more symmetric (dominant vs. non-dominant) than those of soccer players. Another research article by Markou et al. [19] analyzed flexion/extension strength in the lower limbs, as well as internal/external rotation strength in the upper extremities. They used Cybex II+ and found a significant asymmetry between dominant and non-dominant upper limb strength (PT), but not for the lower extremities. Furthermore, they stated that this phenomenon occurred especially among volleyball players who occupied offensive positions.

Moreover, isokinetic testing is a great source of information on muscle strength deficiencies, which is vital in determining the quality of jumps and the risk of joint injury.

Therefore, this study evaluated the level of muscle strength by using isokinetic and isometric measurements—more specifically, the force ratio between the knee flexors and extensors (the values of the torques). Additionally, in order to evaluate the dynamic stabilization of the knee, the strength indexes of these muscles were assessed.

## 2. Materials and Methods

### 2.1. Study Design

This study was a laboratory experiment with a designated division into groups. The strength measurements were performed in two independent sessions during the mid-point of the volleyball season of the Polish League (February). The first session included the measurements of the concentric isokinetic torque of the hamstrings and quadriceps. In addition, on the day before the force measurements, height and body mass were measured. During the second session (2nd day), the isometric muscle strength of the knee extensors (KEs) that developed in a closed kinetic chain (CKC) was examined. The participants were instructed regarding the procedures of the isokinetic and isometric tests and several repetitions were performed for familiarization.

### 2.2. Subjects

Fourteen elite male volleyball players in the division-one Polish Volleyball League (KS Poznan) and 14 male physical education students participated in the experiment. All of the participants were healthy. The volleyball team consisted of nine players who played in offensive positions (two middle blockers, three receivers, and four attackers) and five players who played in defensive positions (three setters and two liberos). The volleyball players were qualified for league matches by the appropriate league medical services. The main division criterion was participation in a high level of volleyball performance (at least five years of competitive experience: 7.5 ± 1.04 years) for the players and non-practice in any sport for the control group (the students). An additional criterion was that all participants were free from any injuries in the lower extremities that could affect their strength performance at the time of testing.

### 2.3. Ethics Statement

The review board approved this experiment. The Bioethical Commission of the Poznan University of Medical Sciences (No 203/08) approved the study design. The procedures were in accordance with the Code of Ethics of the World Medical Association (Helsinki Declaration of 1964). Before signing the informed consent forms, the participants were informed about the experiment’s aim and the risk of injury.

### 2.4. Strength Measurement Procedures

The isokinetic testing was performed at the mid-point of the volleyball season by utilizing the Biodex System 3. In order to obtain the maximum precision and the highest reliability, the tests were conducted in the laboratory of the Department of Biomechanics at the Poznan University School of Physical Education. Before each series of tests, both groups performed a warm-up by exercising for 10 minutes on a MONARK Ergomedic 874E bicycle ergometer. Moreover, during the testing procedure, to maintain optimum readiness between tests, the participants used a Pliant Centauri Omega electric treadmill as a warm-up device. Each test was administered in groups of 3 or 4 persons in order to provide adequate rest between trials. Three angular velocities (ω) were used for the isokinetic tests: 60, 180, and 300 °/s. The range of knee joint motion throughout each measurement was fixed at 90° for all participants. The initial position in which the leg was fully extended was set as 0°. Before every test, participants performed three sub-maximal flexion/extension cycles. The participants then repeated the flexion/extension cycle five times with the goal of generating the maximum torque. The participants performed with their upper limbs crossed and resting on their chest. In order to isolate the work of the muscle groups acting on the knee joint, the torso, pelvis, thigh, and lower leg were stabilized each time. Additionally, a static test was performed (the knee-bend angle was 90°/s) to determine the maximum values of the moments of the forces generated by the examined muscle groups. Measurements of the left and right lower limbs were performed during both the static and isokinetic studies.

### 2.5. Statistical Analysis

Descriptive statistics (the mean and SD) were calculated for all dependent variables. The Shapiro–Wilk test was used to assess the conformity of the statistical distributions of the analyzed variables with a normal distribution. The Mann–Whitney U test for two independent samples and the Wilcoxon signed-rank test for two related samples were used to examine the differences between the groups. Effect sizes were evaluated by calculating Cohen’s d with 95% confidence intervals. Cohen suggested that d + 0.2 be considered a small effect size, 0.5 be considered a medium effect size, and 0.8 be considered a large effect size. The statistical power was determined to be 0.90 at the 0.05 alpha levels.

## 3. Results

The results of the experiments with the twenty-eight participants who were divided into two groups—fourteen volleyball players and fourteen physical education students—were analyzed. The results of the Shapiro–Wilk test showed that the variables were not normally distributed (*p* < 0.05).

The somatic parameters within the experimental group and the control group—given as arithmetic means and standard deviations—had the following values, respectively: height, 195.7 (±6.2) and 178.7 (± 6.7) cm; mass, 87.8 (±6.9) and 73.4 (±8.9) kg; BMI, 22.9 (±1.3) kg/m^2^, which was similar in both groups. The average ages within the experimental group and the control group amounted, respectively, to 23.9 (±3.8) and 23.6 (±1.9) years. A Mann–Whitney U test revealed a significant difference (*p* < 0.05) between the somatic characteristics of the two groups. The volleyball players were taller and heavier than the participants from the control group by 8.7% and 16.4%, respectively. This can be explained by the fact that the volleyball players were a carefully selected group of males with respect to height. Accordingly, their body mass was also greater. However, the age and BMI of both groups were similar.

The results of the PT tests presented in Table 1 indicate that there were statistically significant differences between the two groups in terms of the PT of the knee joint extensors and flexors (*p* < 0.05). One exception was for the PT of the extensors of the right limb at ω = 60 °/s. The volleyball players achieved higher PT values than the control group for all conditions. For the extensors, the differences ranged from 17.1% (right limb at ω = 60 °/s) to 26.2% (right limb at ω = 300 °/s), whereas in the flexor group, the difference ranged from 27.7% (right limb at ω = 180 °/s) to 32.0% (left limb at ω = 60 °/s).

In terms of the PT, the dominant limb was the right one. Except for the ω = 60 °/s test in the volleyball players, the knee extensors in the right limb were slightly stronger, reaching a maximum difference of about 3%. Table 1 also provides information about the values of the joint angle at which the PT was generated. For the experimental group, there was a statistically significant difference between the extensor muscles of the left and right limbs at ω = 180 °/s (*p* < 0.05). There were statistically significant differences between the two tested groups in terms of the joint angle at which the knee joint flexor PT was generated (*p* < 0.05), except for the right limb at ω = 180 °/s and at ω = 300 °/s. The analysis of the isokinetic measurements of the knee joint extensors in both groups shows that the angle at which the PT value was obtained fluctuated between of 56.0° and 62.8°, and the differences between the two groups ranged between 1.9% and 5.1%. The range of motion for all measurements was 90° with a full extension equal to zero. The PT for the extensors for both the players and the control group occurred at an average knee joint angle of 59°, or approximately 34.4% of the range of motion. In the case of the isokinetic measurements of the knee joint flexors, in both groups, the angle at which the PT value was achieved fluctuated within the range of 40.0° to 86.6° of the bending angle, or approximately 4.6% and 31.4% of the range. However, the differences were within a wider range of 4.6% to 31.4%.

The static test results are shown in Table 2. The analysis revealed that the PT values of the extensors (L, R) and flexors (L) were significantly higher in the experimental group than in the control group (*p* < 0.05). For the extensor muscle group, the control group produced 249.1 Nm of torque, while the athletes produced 315.1 Nm—an increase of 26%. For the flexor muscle group, the control group produced 112 Nm and the athletes produced 143.3 Nm—an increase of 30%. When corrected for body weight, however, the difference in favor of the athletes was only 6%.

**Figure 1:**
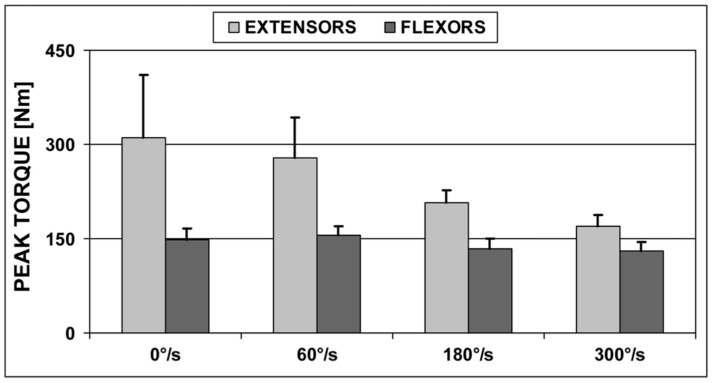
Changes in the peak torque of the right limb as a function of the angular velocity of the knee joints of players.

Figure 1 presents the relationship of PT values generated by extensors and flexors with the angular velocity of the knee joint, which was determined during isokinetic concentric movement tests and the static tests on the players. The higher the contraction velocity was, the lower the tension in the muscle was. The flexor group followed the same pattern, but it was not as pronounced. Unexpectedly, the PT of the flexors when tested statically was lower than the PT in the concentric isokinetic measurements at ω = 60 °/s.

The analysis of the results from Table 3 shows that at ω = 60 °/s, there was a statistically significant difference in the H/Q ratio between the left and right limbs of the athletes (*p* < 0.05). In addition, the H/Q ratio of the players was higher than that of the control group under both conditions. At ω = 60 °/s, the H/Q ratio of the athletes was 17.3% higher than that of the control group, and it was 5% higher under the static conditions.

## 4. Discussion

The main objective of this study was to evaluate the level of muscle strength through isokinetic and isometric measurements—in particular, the force ratio between the knee flexors and extensors (values of the torques). This evaluation will help in the assessment of aspects of the quality of jumps and, thus, in the determination of the load on the volleyball players’ knee and their injury prevention status. Therefore, to confirm the validity of the aim of this research, it seems interesting to conduct a discussion in two interrelated areas. One area concerns the training load as an interpretation of the improvement of a volleyball player’s motor skills and game effectiveness, and the other area is the protection of volleyball players against injuries resulting from the application of the above-mentioned training load.

The correct assessment of the application of the training load in specialized volleyball training, which consists of many components, is mainly based on the level of strength and technical skills. A certain level of strength is necessary in order to carry out training to improve the power of the lower limbs—plyometrics [24]. The jumping skills combined with the technical skills of volleyball players guarantee high performance [24]. Therefore, the assessment of the strength level concerns the muscles of the flexors and extensors of the knee joint, is mainly isokinetic, and is based on torque–angle representations (Alexandre et al. [25]. Polish volleyball players achieved significantly higher isokinetic PTs than those of Greek players. The H/Q ratio in the current study was about 83% of the standard value reported by Coombs et al. [26]. This was higher than that of the Brazilian junior team by 7.1% and lower than that of Greek volleyball players by 16.9% [19,22].

With the knee extensor muscle group, we observed that as angular velocity increased, the PT decreased. This result was in accordance with the theory of A.V. Hill, who stated that the higher the load applied to the muscle is, the lower the contraction velocity will be. Based on this statement, we found some similarities and differences in the static PT values and H/Q ratios that we measured with respect to data found in the literature. With respect to the right knee extensors and flexors, the Polish players produced 31% and 3% higher torque than the group studied by Janiak et al. [27], as well as 9.5% and 32% higher torque than other athlete males of a similar age [28]. The H/Q ratio calculated for the PT of the right limb in the static tests [27]. The H/Q ratio calculated for the PT values of the right limbs (in the static tests) was 0.47 for the players in the current study and 0.34 for the more prestigious Polish national team [27]. Compared to the data provided by Freedson et al. [29], the volleyball players in the current study generated higher PT values in the knee flexors and extensors. The one exception was the PT of the flexors. That value was only slightly higher. Additionally, in the isokinetic tests conducted on the Greek volleyball team, players from Poland achieved 18.2% higher PT values for the knee extensors, but 1.4% lower values for flexors [19]. However, the PT of the flexors of the examined players at the speed of 180 °/s was slightly lower (5.5%) than that of the French national volleyball team. In turn, Pelegrinelli et al. [25] claimed that the isokinetic parameters of the PT, TW, and MP were significantly higher for PRO group volleyball players than for U17 group players in terms of knee extension and flexion (for the knee flexors, only at 120 °/s). Kabacinski et al. [11] compared female volleyball players with basketball players. They showed that the significantly greater H/Q ratio at 60 Ê/s in the volleyball athletes indicated better utilization of the hamstring muscle group in volleyball-specific movements or a weakening of the quadriceps relative to the hamstrings in comparison with the basketball players. Dauty et al. [21], in their research on volleyball players, obtained slightly higher results for the hamstring-to-quadriceps ratio (H/Q)—0.66 vs. 0.60.14—than that indicated by the population norm. They believed that a better indicator of the balance between the knee flexor and extensor in functional conditions would be the ratio measured during the eccentric work of the flexors and the concentric work of the extensors. In turn, Bittencourt et al. [22] conducted tests on Brazilian junior teams and found that the H/Q strength ratio for this group was low at 0.51.15.

In recent years, the successes of male and female Polish volleyball players have inspired the emergence of research projects aimed at biomechanical aspects of dynamic loads in volleyball—namely, traumatogenicity, prevention, and strength training, including nonspecific methods and assessment [30,31,32]. Within the group of the 14 players that were tested, three experienced negative consequences of knee joint injuries. These injuries happened in the past, before the research began. The most serious among them were damage to the lateral meniscus and anterior cruciate ligament or patellar tendinopathy. Increased training volume and exposure to matches can significantly affect modifiable risk factors that are associated with the development of patellar tendinopathy [5]. The data presented by Visnes and Bahr [33] confirm this assumption, as they showed that for each additional hour of training (odds ratio = 1.72) and a set of matches played per week (odds ratio = 3.88), there is a significantly increased risk of injury. This is mainly related to the increased load on the lower limbs due to the increased number of take-offs and landings from different heights after the implementation of specialized volleyball movement structures. According to McNitt-Gray [17], landing from an increased height increases the mechanical demand and the eccentric impact force, which, in turn, has been shown to increase the ankle, knee, and hip extensor moments. Earlier findings by Charlton et al. [5] regarding the increased volume of jumps and the greater vertical displacement of the volleyball player’s body showed that these were also correlated with the presence of patellar tendinopathy [15,34]. The research concerned both volleyball and basketball players. Therefore, the ability to accurately and more effectively monitor loads provides coaching and support staff with the opportunity to avoid training activities that have been shown to increase risk of injury [33,35,36].

From a practical point of view, the results obtained here can be valuable materials for analysis by volleyball coaches and trainers. This experiment showed that the correct isokinetic and isometric assessment of the strength of the knee joint extensors and flexors directly affects the selection of the type of strength training and the load when playing volleyball. Properly selected strength training can improve strength and power production (the application of plyometric training). In turn, from a clinical or physiotherapeutic standpoint, the strength training exercises that are selected should maintain a balance of muscular strength across the knee joints and between opposing muscle groups and, therefore, reduce the frequency of injuries and contribute to a faster recovery from them.

According to this statement, one of the limitations of this study was the fact that four to six weeks of strength training or power training were not included in the preparatory period. This would have allowed changes to be observed in the strength ratio, which would be expressed as the percentage growth of the muscle strength in relation to the measurement before the application of training. Additionally, this would help in the calculation of a strength index that would be defined as the ratio of the maximal torques of the flexors to the maximal torques of the extensors; this would be required for the assessment of the plyometric performance during training [25]. Another limitation would be the small experimental group in this study. However, the athletes were professional volleyball players that represented the club in the highest volleyball league in Poland. It was not possible to recruit additional players from another club, and the main reason was that the club was implementing a different training program during this period.

## 5. Conclusions

In all trials, volleyball players achieved significantly higher peak torque (PT) values for both the extensors and flexors (*p* < 0.05) than those of the control group. However, the strength ratio of the flexors to extensors (H/Q) in the experimental group was only 83% of the standard reported in the literature. There were larger differences in the values obtained in the static tests.

The most developed and dominating muscles in the knee joints of volleyball players are the extensors, accounting for the low strength ratio and dynamic instability of this joint.

Based on a proper assessment of the strength ratio of the knee flexors and extensors, properly selected and implemented resistance training can improve the maximum strength and power production and can reduce the incidence of injuries in volleyball.

## Figures and Tables

**Table 1:** Peak torque of the knee joint extensors and flexors obtained during isokinetic tests with concentric movements (mean ± s) and joint angles at which the peak torque was obtained during these tests (mean ± s).

Parameter		Experimental Group						Control Group					
		ω = 60 °/s		ω = 180 °/s		ω = 300 °/s		ω = 60 °/s		ω = 180 °/s		ω = 300 °/s	
		L	R	L	R	L	R	L	R	L	R	L	R
Extensors PT (N·m)	$\bar{x}$	295.7 ^a^	278.1	201.9 ^a^	206.8 ^a^	163.9 ^a^	169.4 ^a^	223.2 ^a^	230.6	157.2 ^a^	158.3 ^a^	125.1 ^a^	125.1 ^a^
	S	36.1	64.2	20.7	20.0	16.7	17.7	42.9	39.0	30.6	30.0	23.5	23.1
Flexors PT (N·m)	$\bar{x}$	151.2 ^a^	154.8 ^a^	129.7 ^a^	133.5 ^a^	129.6 ^a^	131.1 ^a^	102.8 ^a^	110.4 ^a^	92.4 ^a^	96.5 ^a^	92.1 ^a^	92.4 ^a^
	S	16.3	14.8	18.0	16.6	12.9	13.1	26.0	26.5	14.6	17.4	17.1	15.1
Extensors Angle of PT (°)	$\bar{x}$	61.8	63.8	55.2 ^b^	59.5 ^b^	57.5	54.4	57.1	62.1	55.5	57.0	56.8	58.8
	S	6.1	8.0	6.1	7.0	11.4	18.8	6.8	5.4	4.9	7.3	15.8	13.7
$\bar{x}$ for L&R		62.8		57.4		56.0		59.6		56.3		57.8	
Flexors Angle of PT (°)	$\bar{x}$	42.3 ^a^	37.6 ^a^	74.1 ^a^	75.4	82.6 ^a^	82.6	57.6 ^a^	59.0 ^a^	86.3 ^a^	84.7	87.5 ^a^	85.4
	S	17.0	7.3	17.1	17.1	6.7	6.5	22.0	22.0	7.5	5.2	5.9	6.2
$\bar{x}$ for L&R		40.0		74.8		82.6		58.3		85.5		86.6	

^a^: Mann–Whitney U test, *p* < 0.05. ^b^: Wilcoxon test, *p* < 0.05.

**Table 2:** Peak torques of the knee joint extensor and flexor obtained during a static test with a knee joint angle of α = 90° (mean ± s).

Parameter		Extensors				Flexors			
		Experimental Group		Control Group		Experimental Group		Control Group	
		L	R	L	R	L	R	L	R
PT (N·m)	$\bar{x}$	319.6 ^a^	310.5 ^a^	245.1 ^a^	253.6	138.9 ^a^	147.7 ^a^	108.0 ^a^	116.0
	SD	51.0	100.5	64.8	60.3	19.1	18.0	25.0	24.4
	d	0.13		0.20		0.47		0.46	
$\bar{x}$ for L&R		315.1		249.4		143.3		112.0	
SD		75.5		62.5		15.55		24.7	
d		0.14				0.99			

^a^: Mann–Whitney U test, *p* < 0.05.

**Table 3:** Flexor/extensor ratio obtained from an isokinetic test with concentric movements (ω = 60 °/s) and a static test (α = 90°) (mean ± s).

Parameter		Experimental Group				Control Group			
		ω = 60 °/s		isom α = 90°		ω = 60 °/s		isom α = 90°	
		L	R	L	R	L	R	L	R
F/E (%)	$\bar{x}$	51.3 ^b^	58.4 ^b^	44.2	52.6	46.1	47.5	45.7	46.5
	SD	4.6	13.7	7.5	18.3	8.9	7.1	10.6	7.4
	d	0.69		0.06		0.14		0.08	
$\bar{x}$ for L&R		54.9		48.4		46.8		46.1	
SD		9.15		12.9		8.0		9.0	
d		0.10				0.06			

^b^: Wilcoxon test, *p* < 0.05.

## Data Availability

Data supporting reported results can be found, at Physiotherapy Department, Stanisław Staszic University of Applied Sciences in Piła, info: puss.pila.pl.
